# Early Intervention Effects and Risk Factors for Residual Skin Changes in Infantile Hemangiomas

**DOI:** 10.1111/1346-8138.70388

**Published:** 2026-07-16

**Authors:** Kayoko Okuda, Motoki Nakamura, Reiko Nakamura, Sachiyo Takagi, Akimichi Morita

**Affiliations:** ^1^ Department of Geriatric and Environmental Dermatology Nagoya City University Graduate School of Medical Sciences Nagoya Japan; ^2^ Anti‐Aging Laser Care Center, Division of Dermatology Nagoya City University Mirai Kousei Hospital Nagoya Japan

**Keywords:** early treatment, infantile hemangioma, pulsed dye laser, residual skin changes

## Abstract

Infantile hemangioma (IH) characteristically progresses through proliferative, involuting, and involuted phases, and typically undergoes spontaneous regression after the age of five. Therefore, the traditional management strategy has been based on a “wait‐and‐see policy”. However, the degree and tempo of proliferation vary considerably among individuals, and rapid enlargement can lead to functional impairment or ulceration. Moreover, in some cases, significant residual skin changes may persist even after involution. In Japan, pulsed dye laser (PDL) therapy was approved for insurance coverage in 1996, and early intervention during the proliferative phase has since become the standard approach to prevent such complications. At our institution, we have also actively implemented early PDL therapy, with oral propranolol added in selected cases. We conducted a retrospective analysis of 92 patients with 107 IH lesions who initiated PDL therapy at our dermatology department between January 2014 and December 2022. Earlier initiation of laser treatment was significantly associated with an earlier peak in tumor size (*r* = 0.80, *p* < 0.0001). Furthermore, evaluation of residual skin changes after treatment completion revealed that the nodular type exhibited significantly more severe residual skin changes than the other morphological subtypes (Fisher's exact test, *p* < 0.0001). A peak size ≥ 3 cm^2^ was also identified as a risk factor for severe residual skin changes (multivariate logistic regression analysis, odds ratio 21.6, 95% confidence interval 2.93–358, *p* = 0.0090). In contrast, low birth weight and prematurity were not associated with an increased risk of severe residual skin changes when laser treatment was initiated. In conclusion, early PDL initiation was associated with earlier peak lesion size in IHs. Nodular‐type lesions and peak size ≥ 3 cm^2^ predicted severe long‐term residual skin changes, supporting early risk stratification and timely consideration of systemic therapy in high‐risk cases.

## 1 Introduction

Infantile hemangioma (IH) is a benign tumor arising from vascular endothelial cells and is classified as a vascular tumor by the International Society for the Study of Vascular Anomalies (ISSVA). It is one of the most common tumors in infancy and occurs more frequently in girls, premature infants, and low‐birth‐weight infants, irrespective of race. Incidence rates vary by ethnicity, with a reported incidence of 0.8%–1.7% in the Japanese population [1, 2].

Infantile hemangioma (IH) demonstrates a characteristic natural history distinct from that of typical tumors. At birth, lesions are usually absent or appear only as subtle erythematous papules or small precursor lesions composed of telangiectasia. Lesions typically become apparent around 2 weeks after birth and subsequently progress through proliferative, plateau, and regression phases, with regression nearly complete by the late resolution phase. Recent studies have reported that untreated IHs reach approximately 80% of their peak size by 3 months of age [3], with particularly rapid enlargement occurring between 5.5 and 7.5 weeks of age [4]. Growth then continues gradually until approximately 5 months of age [3], at which point lesions typically reach their peak. After the plateau phase, regression begins between 6 and 12 months of age [5] and is usually complete by 3.5–4 years [6, 7]. However, a follow‐up study of 137 untreated IH lesions in 97 patients in the Netherlands reported residual skin changes in 69% of cases [6], with superficial IHs demonstrating significantly more residual changes (74%) than deep IHs (25%) [6].

Traditionally, the clinical approach to IH has been conservative observation, based on the expectation of spontaneous resolution. This has been widely accepted among physicians caring for IH, including pediatricians and dermatologists. However, one study reported that among untreated lesions, 87% ultimately required some form of intervention for residual skin changes, and 76.5% underwent surgical excision [7], raising awareness of the need for early treatment aimed at preventing such sequelae.

Historically, active intervention was reserved for severe cases associated with life‐threatening complications, functional impairment, or ulceration and bleeding. For many years, oral or local corticosteroid injections served as the primary treatment option. Subsequently, with the development of pulsed dye laser (PDL) technology for vascular lesions, PDL therapy gained insurance coverage in Japan in 1996, establishing early laser intervention as a mainstream treatment approach. In 2016, propranolol hydrochloride syrup also became covered by insurance, becoming the first‐line therapy for IH with life‐threatening or functionally compromising complications, ulcerated lesions, rapidly proliferating IH, lesions on exposed areas such as the face, and tumor‐type IH. This expanded the ability to treat lesions previously difficult to manage with laser monotherapy due to limited penetration depth.

At our institution, early initiation of laser therapy has also become increasingly common in recent years, and in selected cases, treatment is initiated as early as the neonatal period in collaboration with the neonatal intensive care unit (NICU). This study aimed to evaluate the impact of early treatment initiation on the clinical course of IH and to identify predictors of severe residual skin changes.

## 2 Methods

### 2.1 Patient Cohort

We retrospectively evaluated 92 patients (26 males and 66 females) with infantile hemangioma who were diagnosed and initiated pulsed dye laser (PDL) therapy at Nagoya City University Hospital between January 2014 and December 2022, encompassing 107 lesions. Clinical photographs obtained at the initial consultation, at each laser treatment session, and during follow‐up after completion of therapy were used for assessment. Lesions were classified into four morphological types: superficial type (elevation ≤ 3 mm), mass type, subcutaneous type, and mixed type. This study was approved by the Nagoya City University Clinical Research Review Board (Approval No. 60‐24‐0122).

### 2.2 Laser Therapy

Laser irradiation was performed using the Vbeam (Candela Corporation, Wayland, Massachusetts, USA), a 595 nm PDL. Irradiation parameters included a spot size of 7 mm, pulse width of 1.5–20 ms, and output of 8–12.5 J/cm^2^. Treatments were performed at 1–1.5 month intervals until peak response was confirmed, followed by intervals of 3 months or longer. Treatment was discontinued when regression had progressed to the point where additional PDL irradiation was deemed ineffective.

### 2.3 Oral Propranolol Therapy

Since 2016, oral propranolol therapy has been added for severe cases meeting indications, provided there were no contraindicating complications and informed consent was obtained from the family. Treatment was initiated after 5 weeks of postnatal age, or after five corrected weeks of gestation for preterm infants. Initiation was conducted under 5 days of inpatient monitoring by pediatricians at Nagoya City University Hospital. Treatment was started at 1 mg/kg/day, increased by 1 mg/kg/day at intervals of 2 days or more, and maintained at 3 mg/kg/day. Treatment was discontinued or tapered off upon achieving sufficient tumor reduction.

### 2.4 Evaluation of Peak Out

The age at which a clear regression trend was first observed in serial clinical photographs was defined as the peak‐out age.

### 2.5 Evaluation of Residual Skin Changes

**FIGURE 1:**
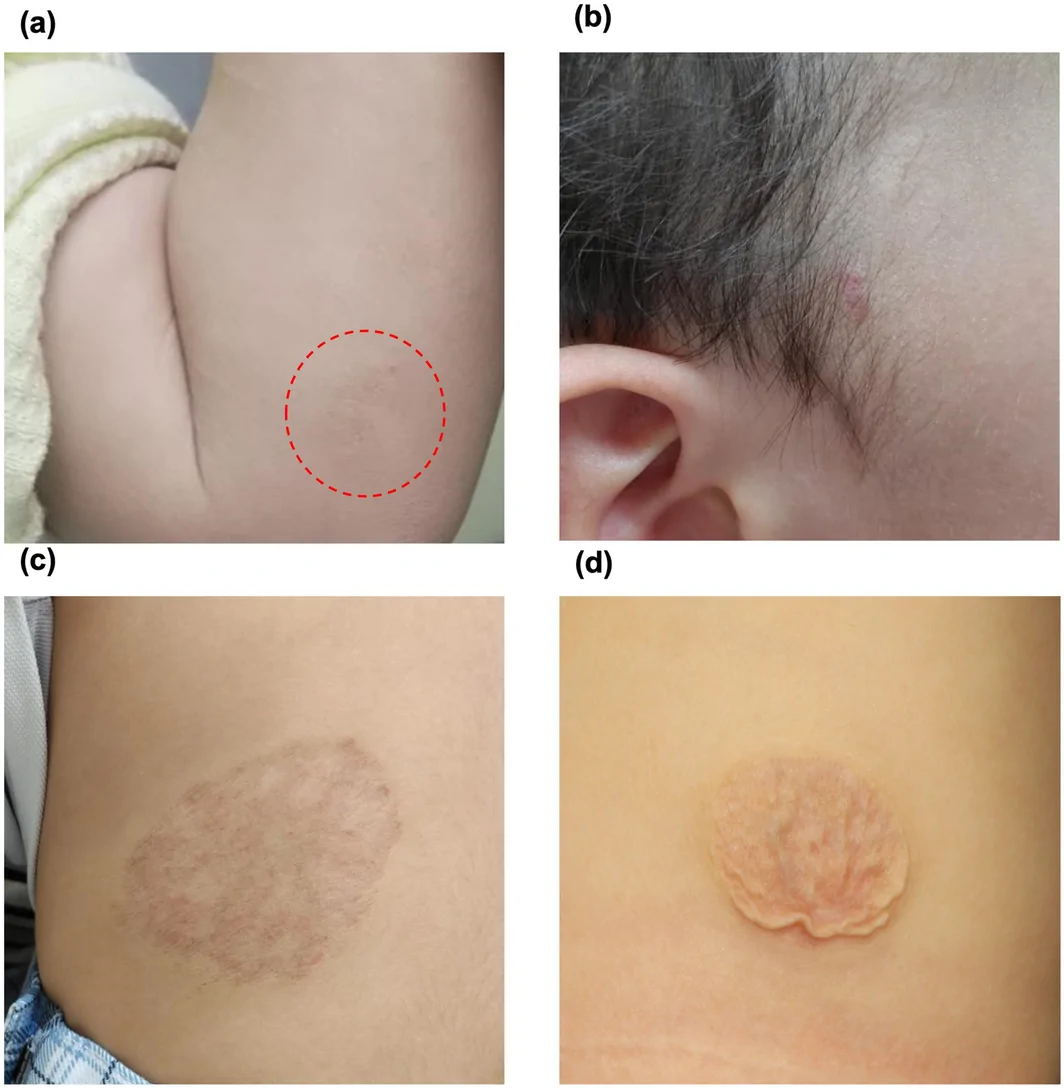
Evaluation of residual lesions. Lesions evaluated were those present at least 1 year after treatment completion, or those with only minor telangiectasia or erythema present at least 6 months after treatment completion. The observation period extended until March 2025. For the 87 lesions for which follow‐up was possible to meet the criteria, two dermatologists evaluated residual lesions on a four‐point scale (none, mild, moderate, severe). (a) None. (b) Mild. Residual slight erythema or telangiectasia. (c) Moderate. Residual erythema, pigmentary changes (hyperpigmentation or hypopigmentation), or mild elevations or skin atrophy. (d) Severe. Residual marked pigmentary changes, skin laxity, or skin atrophy.

Lesions assessed were those present more than 1 year after treatment completion, or those with only minor telangiectasia or erythema present more than 6 months after treatment completion. The observation period extended until March 2025. For the 87 lesions meeting these criteria, two dermatologists evaluated residual changes on a four‐point scale (none, mild, moderate, severe) (Figure 1). Mild: slight erythema or telangiectasia. Moderate: erythema, pigmentary changes (hyperpigmentation or hypopigmentation), or mild elevation or skin atrophy. Severe: marked pigmentary changes, skin laxity, or skin atrophy.

### 2.6 Statistical Analysis

Statistical analysis was performed using GraphPad Prism 10 (GraphPad Software, San Diego, CA). The Kruskal–Wallis test was used to assess differences among the three groups within each morphological type. When significant differences were observed, Dunn's multiple comparison test was performed. Simple linear regression analysis was performed to evaluate the association between age at treatment initiation and each clinical indicator. To compare residual changes, Fisher's exact test was used to evaluate overall statistical significance. When significant results were obtained, residual analysis was performed. For major contributing categories, 2 × 2 contingency tables were created and individual Fisher's exact tests were conducted. Multivariate logistic regression analysis was performed to identify risk factors of residual changes. A *p* < 0.05 was considered statistically significant for all tests.

## 3 Results

### 3.1 Patient Characteristics

From January 2014 to December 2022, laser treatment was initiated in 92 patients with 107 lesions (Tables 1 and 2). The male‐to‐female ratio was 28.3% male and 71.7% female, showing a clear predominance of females. Nearly half were full‐term infants with normal birth weight. Among those for whom this information was available, 20.7% were preterm infants and 23.9% were low‐birth‐weight infants; there were no macrosomic infants. According to Japan's Ministry of Health, Labour and Welfare's vital statistics, the recent proportion of low‐birth‐weight infants in Japan is approximately 9.4%. Most cases were solitary lesions, with 12 patients presenting with multiple lesions. The largest multiple‐case involved 29 lesions and was complicated by an intrahepatic hemangioma. Lesion size was < 3 cm^2^ in 65.4% of cases, and the most common type was superficial (73.8%). The most frequent sites of occurrence were the head/neck and trunk. Treatment involved PDL alone for 79 lesions and combined with oral propranolol for 28 lesions.

**TABLE 1:** Details of 92 IH patients who underwent PDL treatment.

Patients *n* = 92	
Sex	
Male	26 (28.3%)
Female	66 (71.7%)
Gestational age	
Term (37–42 weeks)	45 (48.9%)
Preterm (< 37 weeks)	19 (20.7%)
Post term (> 42 weeks)	0 (0%)
Unknown	28 (30.4%)
Birthweight	
Normal (2500–3999 g)	44 (47.8%)
< 2500 g	22 (23.9%)
≧ 4000 g	0 (0%)
Unknown	26 (28.3%)
Number of IH per infant	
*n* = 1	80 (87.0%)
*n* = 2	9 (9.8%)
*n* = 4	1 (1.1%)
*n* = 6	1 (1.1%)
*n* = 29	1 (1.1%)

**TABLE 2:** Details of 107 IH lesions treated with PDL therapy.

Lesions *n* = 107	
Size of IH	
< 3 cm^2^	70 (65.4%)
> 3–10 cm^2^	24 (22.4%)
> 10–30 cm^2^	10 (9.3%)
> 30 cm^2^	3 (2.8%)
Morphological type	
Superficial type	79 (73.8%)
Mass type	21 (19.6%)
Subcutaneous type	0 (0%)
Mixed type	7 (6.5%)
Lesion site	
Head and neck	37 (34.6%)
Trunk	41 (38.3%)
Vulvar‐perineal‐gluteal region	8 (7.5%)
Upper limb	7 (6.5%)
Hand	5 (4.7%)
Lower limb	6 (5.6%)
Feet	3 (2.8%)
Distribution of 37 head and neck IH	
Capillitium	13 (35.1%)
Forehead/temple	8 (21.6%)
Eye/periocular	2 (5.4%)
Nose	0 (0%)
Cheek	3 (8.1%)
Lips/philtrum	4 (10.8%)
Ears	3 (8.1%)
Chin	1 (2.7%)
Neck	3 (8.1%)
Treatment	
PDL only	79 (73.8%)
PDL + Oral propranolol	28 (26.2%)

### 3.2 Treatment Course

Among the 107 lesions treated with laser therapy, treatment was initiated during the proliferative phase (before the peak‐out age) in 96 lesions. Furthermore, 102 lesions completed treatment at our institution without interruption or loss to follow‐up, including discontinuation due to relocation. Therefore, age at peak‐out and number of treatment sessions until peak‐out were evaluated in 96 lesions, whereas age at end of treatment and the total number of treatments were evaluated in 102 lesions.

**FIGURE 2:**
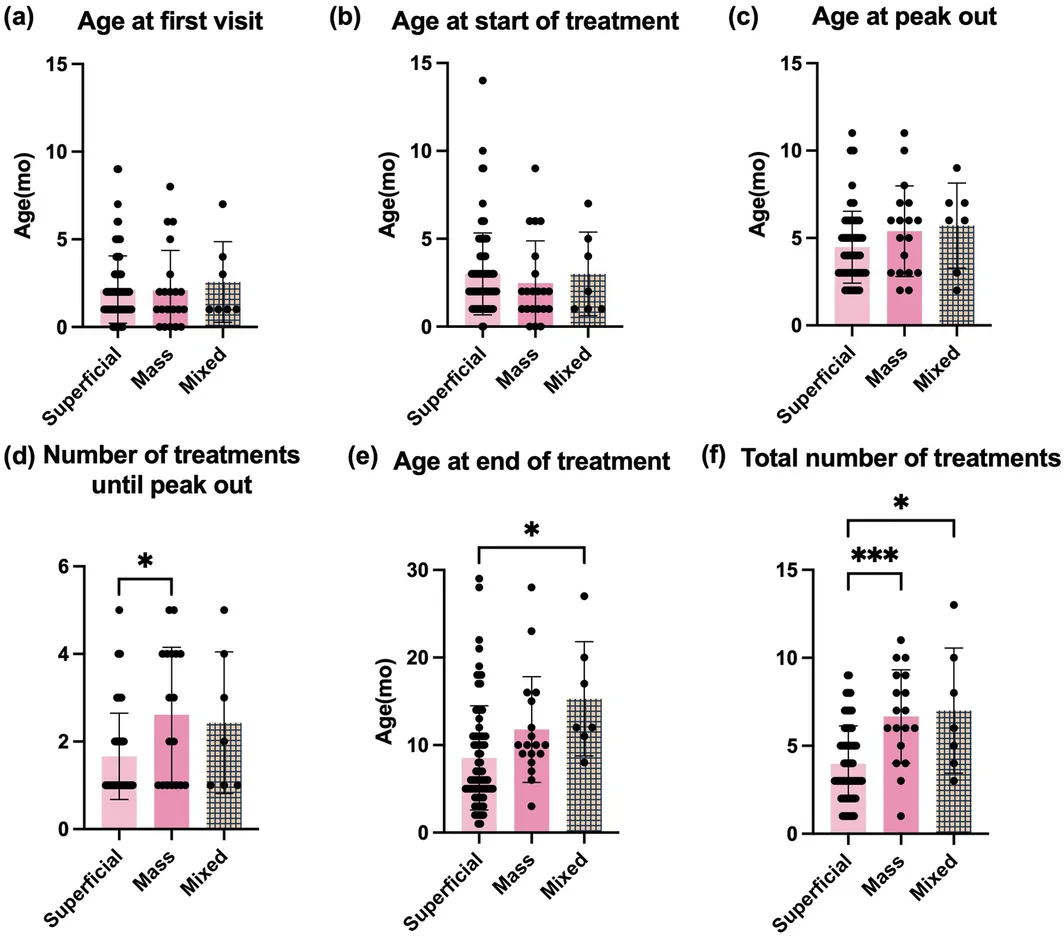
Details of the treatment course for each morphological type. (a–c) The mean age at first visit was 2.2 months, the mean age at start of treatment was 2.9 months, and the mean age at peak out was 5.1 months. No significant differences were observed between morphological types for any of these parameters. (d) The number of treatment sessions until peak out was 1.7 for the superficial type, 2.6 for the mass type, and 2.4 for the mixed type. The mass type required significantly more treatment sessions than the superficial type (*p* = 0.043). (e) The age at end of treatment was 8.5 months for the superficial type, 11.8 months for the mass type, and 15.3 months for the mixed type. There was a significant difference between the superficial type and the mixed type (*p* = 0.011). (f) The total number of treatment sessions was 4.0 for the superficial type, 6.7 for the mass type, and 7.0 for the mixed type. Compared to the superficial type, the mass type (*p* = 0.0005) and the mixed type (*p* = 0.049) had significantly more treatment sessions. *p < 0.05, ***p < 0.001.

The mean age at first visit was 2.2 months, the mean age at treatment initiation was 2.9 months, and the mean age at peak out was 5.1 months, with no significant differences between morphological types (Figure 2a–c). The number of treatment sessions until peak out was 1.7 for the superficial type, 2.6 for the mass type, and 2.4 for the mixed type. The number of sessions was significantly higher for the mass type compared to the superficial type (*p* = 0.043) (Figure 2d). The age at end of treatment was 8.5 months for the superficial type, 11.8 months for the mass type, and 15.3 months for the mixed type, with a significant difference between the superficial type and the mixed type (*p* = 0.011) (Figure 2e). The total number of treatment sessions was 4.0 for the superficial type, 6.7 for the mass type, and 7.0 for the mixed type. Compared to the superficial type, the mass type (*p* = 0.0005) and the mixed type (*p* = 0.049) required significantly more sessions (Figure 2f).

### 3.3 Relationship Between Age at Start of Treatment and Treatment Effectiveness

**FIGURE 3:**
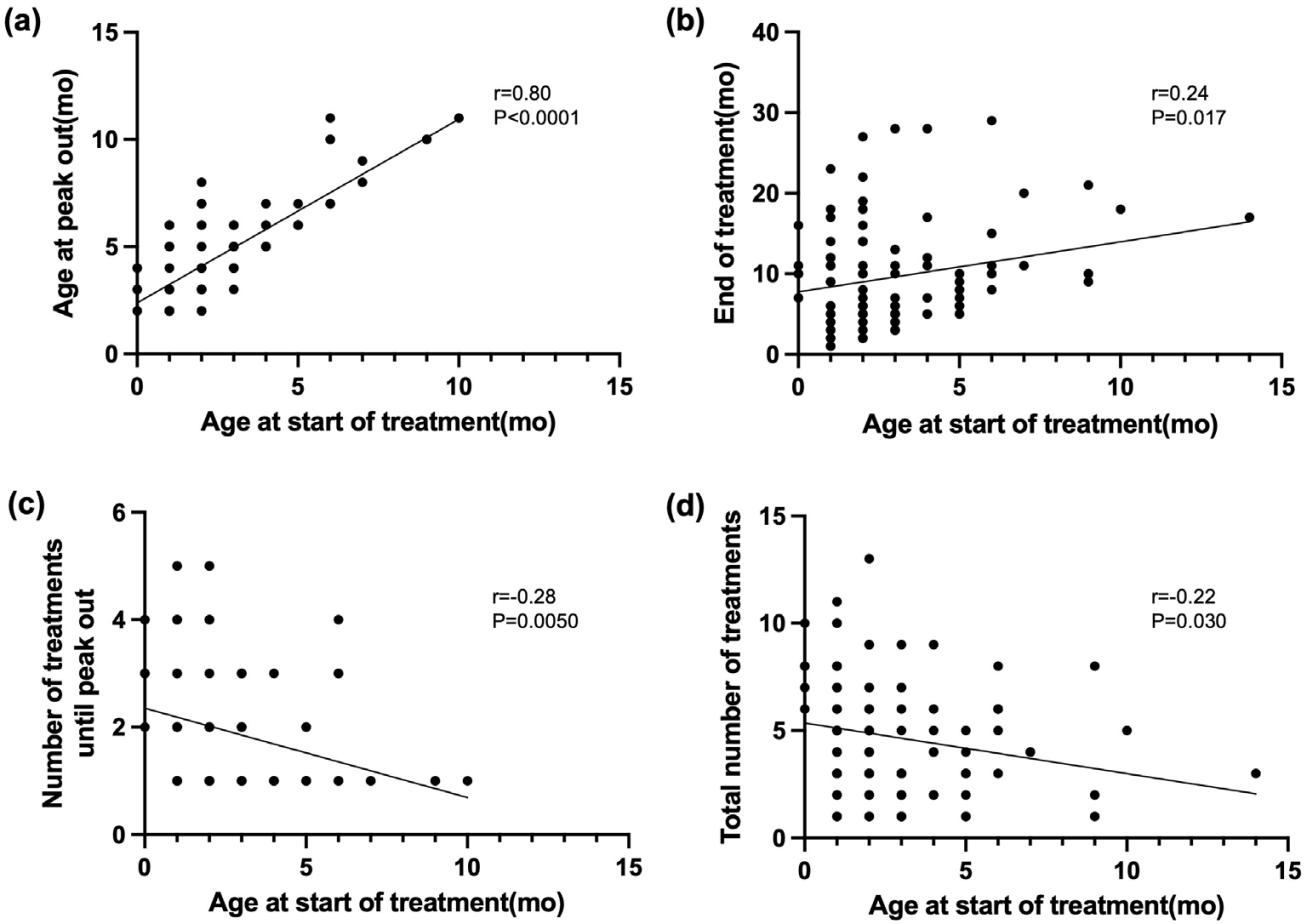
Relationship between age at start of treatment and treatment efficacy. (a) Earlier treatment initiation was associated with earlier peak out (*r* = 0.80, *p* < 0.0001). (b) The age at end of treatment was also earlier when treatment started earlier (*r* = 0.24, *p* = 0.017). (c, d) Regarding the number of treatment sessions, starting earlier tended to increase both the number of sessions until peak out (*r* = −0.28, *p* = 0.0050) and the total number of treatment sessions (*r* = −0.22, *p* = 0.030).

Earlier treatment initiation was associated with earlier peak out (*r* = 0.80, *p* < 0.0001) (Figure 3a). The age at the end of treatment was also earlier with earlier treatment initiation (*r* = 0.24, *p* = 0.017) (Figure 3b). In contrast, earlier initiation tended to be associated with both a greater number of treatment sessions until peak out (*r* = −0.28, *p* = 0.0050) and a greater total number of treatment sessions (*r* = −0.22, *p* = 0.030) (Figure 3c,d). To assess the potential influence of concomitant propranolol therapy, the analysis was repeated in the PDL monotherapy group (*n* = 79). The results were essentially unchanged. Significant positive correlations were observed between the age at treatment initiation and both the age at peak‐out (*r* = 0.81, *p* < 0.0001; Figure S1a) and the age at treatment completion (*r* = 0.24, *p* = 0.042; Figure S1b). Significant negative correlations were also found between the age at treatment initiation and the number of treatment sessions until peak‐out (*r* = −0.32, *p* = 0.0073; Figure S1c), and between the age at treatment initiation and the total number of treatment sessions (*r* = −0.28, *p* = 0.016; Figure S1d).

### 3.4 Evaluation of Residual Skin Changes

**FIGURE 4:**
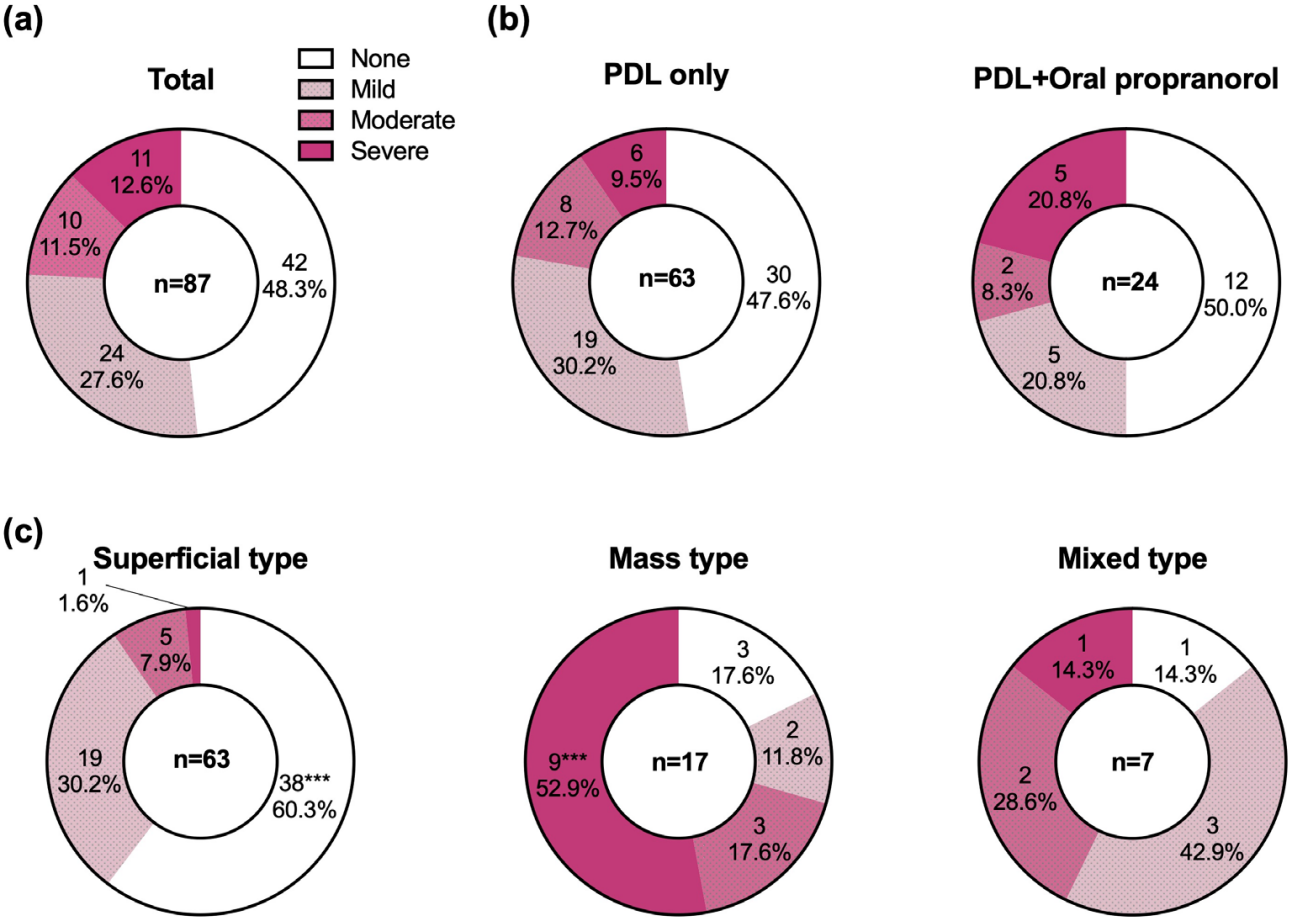
Details of the 87 residual skin changes that could be evaluated during follow‐up after treatment. (a) Overall (*n* = 87). No residual skin changes were present in 42 cases (48.3%), mild residual skin changes in 24 cases (27.6%), moderate residual skin changes in 10 cases (11.5%), and severe residual skin changes in 11 cases (12.6%). (b) Among the 87 lesions, 63 were treated with PDL alone, and 24 were treated with PDL combined with oral propranolol. No statistically significant difference in residual lesion severity was observed between the monotherapy and combination therapy groups (Fisher's exact probability test, *p* = 0.477). (c) Superficial type (*n* = 63), mass type (*n* = 17), mixed type (*n* = 7). In superficial type, no residual skin changes were significantly more common (60.3% of 38 cases, *p* < 0.0001), while in mass type, severe residual lesions were significantly more common (52.9% of nine cases, *p* < 0.0001). The mass type showed a tendency toward a higher incidence of severe residual skin changes compared to other types. ***p < 0.001.

Follow‐up after treatment allowed evaluation of residual skin changes in 87 lesions. Overall, 42 cases (48.3%) showed no residual skin changes, 24 cases (27.6%) showed mild residual skin changes, 10 cases (11.5%) showed moderate residual skin changes, and 11 cases (12.6%) showed severe residual skin changes (Figure 4a). Of these 87 lesions, 63 were treated with PDL alone, and 24 with PDL combined with oral propranolol. No statistically significant difference in the severity of residual skin changes was observed between the monotherapy and combination therapy groups (Fisher's exact probability test, *p* = 0.477) (Figure 4b). However, significant differences were found among morphological subtypes (*p* < 0.0001). In the superficial type, no residual skin changes were observed in 38 cases (60.3%), which was significantly higher (*p* < 0.0001). In the mass type, severe residual lesions were observed in nine cases (52.9%), also significantly higher (*p* < 0.0001) (Figure 4c). The mass type tended to develop severe residual skin changes more frequently than other subtypes.

### 3.5 Examination of Risk Factors for Moderate or Severe Residual Skin Changes

Multivariate logistic regression analysis was performed to identify risk factors for moderate or severe residual skin changes (Table 3). Peak size ≥ 3 cm^2^ (odds ratio [OR] = 21.6, 95% confidence interval [CI]: 2.93–358, *p* = 0.0090) and a higher total number of treatment sessions (OR = 1.79, 95% CI: 1.22–3.17, *p* = 0.0136) were significant risk factors. No statistically significant associations were found for sex, gestational age, birth weight, lesion site, age at treatment initiation, or age at peak out.

**TABLE 3:** Multivariate logistic regression analysis for risk factors. Multivariate logistic regression analysis was performed for risk factors associated with moderate or greater residual skin changes.

	Comparison	Reference	Odds ratio (95% CI)	*p*
Sex	F	M	2.08 (0.13, 74.3)	0.6284
Gestational age			0.96 (0.86, 1.06)	0.4219
Birth weight			1.00 (1.00, 1.00)	0.9491
Lesion site	Head & neck	Other sites	0.96 (0.04, 13.6)	0.9746
Peek size	≧ 3cm^2^	< 3cm^2^	**21.6 (2.93, 358)**	**0.0090**
Age at start of treatment			1.37 (0.81, 2.83)	0.3238
Age at peek out			0.95 (0.50, 1.69)	0.8771
Total number of treatments			**1.79 (1.22, 3.17)**	**0.0136**

*Note:* Peak size ≥ 3 cm^2^ (odds ratio [OR] = 21.6, 95% confidence interval [CI]: 2.93–358, *p* = 0.0090) and higher total number of treatments (OR = 1.79, 95% CI: 1.22–3.17, *p* = 0.0136) were significant risk factors. No statistically significant differences were observed for sex, gestational age, birth weight, lesion site, age at start of treatment, or age at peak out.

Abbreviation: CI, confidence interval.

## 4 Discussion

In this study, we first examined the importance of early intervention and found that earlier initiation of laser therapy was associated with a more rapid achievement of peak lesion involution. Evaluation of residual skin changes after treatment revealed that mass‐type lesions and those with a tumor size of ≥ 3 cm^2^ were more likely to result in cosmetically significant residual skin changes. Although infantile hemangioma tends to occur more frequently in preterm and low‐birth‐weight infants, these factors themselves were not identified as risk factors for residual skin changes when treatment was initiated appropriately.

Treatment options for IH include PDL and oral propranolol, as used in this study, as well as other modalities such as Nd:YAG laser [8] and intense pulsed light (IPL) [9], and topical β‐blockers such as timolol [10]. Meta‐analyses indicate that topical β‐blockers for superficial IH are equally effective as laser therapy [11] and oral propranolol [12] while causing fewer side effects than oral propranolol [12]. These suggest that topical β‐blockers may become a potential first‐line treatment for superficial IH in the future. Currently, no topical β‐blocker agents are approved for IH in Japan. PDL and oral propranolol remain the mainstream treatment options covered by insurance.

Pulsed dye laser (PDL) devices with a wavelength of 577 nm [13] were developed in the 1980s. Subsequently, wavelengths gradually increased, and 595 nm has recently become the standard. Pulsed dye laser (PDL) is used to treat vascular lesions based on the principle of selective photothermolysis of oxygenated hemoglobin [14]. Current devices offer variable fluence and pulse width, and the incorporation of a dynamic cooling device (DCD) allows safer irradiation even at higher fluences. Optimal parameters for IH remain unclear; however, in Asians, longer pulse widths of 10–20 ms are considered safer and more effective [15]. Another report found no difference in efficacy between short pulses (1.5–3 ms) and long pulses (10 ms) [16]. In this cohort, the 20 ms long pulse was used in most cases, whereas short pulses were selected in lesions with minimal thickness. Nonetheless, pulse width varied during treatment for individual patients depending on timing, making it difficult to evaluate pulse‐width‐specific treatment effects in this study.

The efficacy of oral propranolol for IH was discovered incidentally in 2008 [17]. Propranolol is a non‐selective β₁/β_2_‐adrenergic receptor (β₁‐/β_2_‐AR) antagonist, and its inhibitory effect on IH proliferation is primarily attributed to its antagonistic action on β_2_‐AR [18]. Although oral propranolol has become the first‐line drug for IH, reports indicate a treatment success rate of approximately 60% [19]. Recent studies suggest that β_3_‐AR expression may contribute to treatment‐resistant IH [20].

In recent years, the combination of PDL and oral propranolol, two modalities with different mechanisms of action, has been suggested to enhance treatment efficacy, including higher rates of complete tumor regression [21]. In our cohort, some patients received combination therapy; however, PDL remained the primary modality, and oral propranolol was added selectively based on the degree of tumor proliferation. Accordingly, we evaluated overall clinical outcomes in real‐world practice by assessing the age at peak lesion involution and the severity of residual skin changes.

Previous reports have also examined the safe and optimal timing for initiating PDL therapy. He et al. analyzed 180 cases of IHs treated with PDL, grouped by age at initial visit, and reported that earlier treatment initiation resulted in fewer treatment sessions, with no significant differences between groups in overall efficacy or adverse events [22]. Furthermore, initiating treatment in the neonatal period not only reduces the number of treatment sessions but also enables effective treatment at lower fluence and faster recovery from adverse reactions such as purpura [23]. In this study, earlier treatment initiation similarly led to an earlier peak‐out, and no adverse events occurred with neonatal irradiation. Previous meta‐analyses have also suggested that PDL may shorten the proliferative phase and accelerate regression [24]. To facilitate earlier treatment initiation, it is essential to establish systems that allow rapid access to treatment facilities. Collabotarion and information sharing with local pediatric and dermatology clinics are particularly important.

With therapeutic intervention becoming mainstream, it is important to consider how residual skin changes differ between treated and untreated IH. In a review of 432 cases, Chelleri et al. reported that 71% of patients received at least one treatment (oral steroids, oral propranolol, topical timolol, or PDL). Among untreated patients, 75.2% exhibited residual skin changes, compared with 41.4% of treated patients (*p* < 0.001) [25]. In our cohort, 51.7% of treated patients had mild or greater residual skin changes. As expected, mass‐type lesions showed significantly more severe residual skin changes than superficial‐type lesions. Additionally, lesion size ≥ 3 cm^2^ was identified as a significant risk factor, suggesting that patients with such lesions may benefit from systemic therapy with oral propranolol. The American Academy of Pediatrics guidelines [26] similarly identify high‐risk IH as lesions exceeding 2 cm in diameter in the head and neck region (or > 1 cm in the facial area for infants < 3 months), and lesions > 2 cm or > 2 mm thick on the trunk or extremities. Our findings are consistent with these criteria.

Infantile hemangioma (IH) can proliferate rapidly, sometimes changing visibly from day to day. Families should be informed in advance that mass type lesions and those ≥ 3 cm^2^ carry a higher risk of moderate‐to‐severe residual skin changes. In such cases, clinicians should be prepared to initiate oral propranolol promptly to avoid missing the optimal treatment window. Conversely, preterm birth and low‐birth‐weight were not associated with increased risk when appropriate treatment was provided, which may be reassuring for families.

The results of this study highlight the importance of early intervention in IH and identify risk factors associated with severe residual skin changes. We hope that these findings will contribute to optimizing future treatment strategies and help ensure that patients with IH achieve outcomes with minimal long‐term residual skin changes.

In conclusion, this retrospective study demonstrates that earlier initiation of PDL therapy for infantile hemangiomas is associated with an earlier achievement of peak lesion involution and may contribute to reducing long‐term residual skin changes. Mass‐type lesions and lesions with a peak size of ≥ 3 cm^2^ were identified as significant risk factors for moderate‐to‐severe residual skin changes, whereas prematurity and low birth weight were not associated with increased risk when appropriate treatment was provided.

These findings highlight the importance of early risk stratification and timely therapeutic intervention in infantile hemangiomas. For lesions with high‐risk features, early consideration of systemic therapy in combination with PDL may be warranted to minimize cosmetically significant residual skin changes and optimize long‐term outcomes.

## Funding

This work was supported by the Grant‐in‐Aid for the Outstanding Research Group Support Program of Nagoya City University (Grant Number: 202401102).

## Conflicts of Interest

The authors declare no conflicts of interest.

## Supporting information


**Figure S1:** Relationship between age at start of treatment and treatment efficacy in the PDL monotherapy group (*n* = 79). (a) Earlier treatment initiation was associated with earlier peak out (*r* = 0.81, *p* < 0.0001). (b) The age at end of treatment was also earlier when treatment started earlier (*r* = 0.24, *p* = 0.042). (c, d) Regarding the number of treatment sessions, starting earlier tended to increase both the number of sessions until peak out (*r* = −0.32, *p* = 0.0073) and the total number of treatment sessions (*r* = −0.28, *p* = 0.016).

## Data Availability

The data that support the findings of this study are available on request from the corresponding author. The data are not publicly available due to privacy or ethical restrictions.
